# Four New Species and Six Combinations of *Prunulus* (Mycenaceae) from China

**DOI:** 10.3390/jof12030172

**Published:** 2026-02-27

**Authors:** Rui Wang, Ke Wang, Xiao-Dan Yu, Chang-Lin Shan, Hai-Feng Liu, Di Liu, Tie-Zheng Wei

**Affiliations:** 1 Department of Horticulture and Landscape Architecture, College of Agriculture, Yanbian University, Yanji 133002, China; ruiwangyn@163.com (R.W.); haifengliu@163.com (H.-F.L.); 2 Fungarium, Institute of Microbiology, Chinese Academy of Sciences, Beijing 100101, China; wangk@im.ac.cn; 3 College of Biological Science and Technology, Shenyang Agricultural University, Shenyang 110866, China; yuxd126@126.com; 4 Jilin Forestry Technology Extension Station, Changchun 130022, China; cl88628198@163.com

**Keywords:** *Prunulus*, *Mycena* sect. *Calodontes*, new taxon, taxonomy, phylogeny

## Abstract

Four new species of *Prunulus,* found in China—*P. applanatus*, *P. fulvescens*, *P. fulvipes* and *P. leptocollus* spp. nov.—are described based on morphological and molecular phylogenetic analyses, performed using maximum likelihood (ML) and Bayesian inference (BI) methods on a concatenated dataset of the ITS, *rpb1*, and *tef-1α* gene regions. *Prunulus applanatus* is characterized by a pale grey-brown and applanate pileus with a slight lilac tint, and a distinctly striate margin. *Prunulus fulvescens* is characterized by a pileus fading from dark brown to yellowish-brown. *Prunulus fulvipes* is characterized by a pileus with a brown center and a lighter brownish-white margin, and a longer stipe with a yellowish-brown base. *Prunulus leptocollus* is characterized by pale lilac basidiomata, and cheilocystidia with slightly narrowed necks. Our study provides detailed anatomical illustrations and photographs of fresh basidiocarps from these species. Additionally, six *Mycena* species of sect. *Calodontes*—*M. brunneocystidiata*, *M. rosea*, *M. subbrunnea*, *M. subpura*, *M. variispora*, and *M. violaceardesiaca*—are combined with *Prunulus* based on the results of the phylogenetic analysis.

## 1. Introduction

*Prunulus*, a genus established by Gray in 1821 [1], is characterized by glabrous and purplish-, brownish-, reddish, or pinkish-tinged basidiomata; umbonate and glabrous pilei; mostly amyloid basidiospores; smooth and fusiform to clavate cystidia; and smooth pileipellis and stipitipellis [2,3,4,5,6]. Elucidating the taxonomy of *Prunulus* has long been challenging due to its small basidiomata and the variable coloration of its pileus. Murrill [7] recorded 106 *Prunulus* species that were morphologically similar to *Agaricus denticulatus* (=*Prunulus pelianthinus*). However, as more species were described and more distinctive microscopic characteristics were used for species delimitation, many *Prunulus* species were transferred to *Mycena*, with the type species assigned to sect. *Calodontes* [3,4,8,9]. However, the results of a molecular phylogeny analysis of *Mycena* species based on the LSU gene showed that sect. *Calodontes* forms a well-supported clade distant from the type species of *Mycena* [2].

Recently, new *Prunulus* species (as *Mycena* spp.) have been proposed based on a combination of extensive morphological investigations and molecular methods. For example, Chew et al. [10] described three new species and one new variety of *Prunulus* from Peninsular Malaysia. Cortes-Perez et al. [11] described five new species from Mexico. Nagamune et al. [12] also proposed one new taxa, *P. densilamellatus* (Nagamune, S. Kigawa & N. Endo) K.L. Yang, Jia Y. Lin & Zhu L. Yang (as *M. densilamellata* Nagamune, S. Kigawa & N. Endo), from Japan. Through in-depth field surveys and the application of molecular techniques, fourteen new species have been discovered in mixed coniferous–broadleaf forests across northeastern, central, and northern China [6,13,14,15,16,17].

In a survey of macrofungi in China, four undescribed species of *Prunulus* were found based on morphological and phylogenetic analyses, and were illustrated with photos of their morphological and microscopical characteristics. Presently, eighteen new *Prunulus* species, including the four in this study, are described from China. Our work expands on the documented diversity of *Prunulus* in China and also globally.

## 2. Materials and Methods

### 2.1. Specimens Collected and Morphological Description

The specimens used in this study were collected from Liaoning, Hebei, Beijing, Shanxi, Anhui, and Sichuan, China. Detailed morphological characteristics and collection-related data of fresh basidiomata were recorded after capturing photographs of their habitats with a digital camera. The specimens were then dehydrated in an oven at 45 °C. All specimens were deposited in Fungarium, Institute of Microbiology, Chinese Academy of Sciences (HMAS).

Thin sections were prepared from tissues of mature fruiting bodies, treated with 5% KOH, and stained with 1% Congo red. Melzer’s reagent was used to detect amyloid in the basidiospores [18]. The shape and size of the basidiospores, basidia, cystidia, lamellae trama, and pileipellis were recorded in detail using a Nikon Eclipse Ni-U microscope (Nikon Corporation, Tokyo, Japan). Images were captured using Image Analysis System 11.0. At least 30 basidiospores and 20 basidia were measured. The following formulae were used to assess the basidiospores: (a) b–c (d) × (e) f–g (h) μm, [Q = i–j, Q_m_ = k ± m]. Here, (a) and (d) represent minimum and maximum length (5% extremum); b–c and f–g represent 90% of the measured length and width, respectively; Q values i–j have similar meanings; and k ± m denotes the mean Q value of all basidiospores ± sample standard deviation [6,19].

### 2.2. DNA Extraction and PCR Amplification

Genomic DNA was extracted from the specimens using NuClean Plant Genomic DNA Kit (Jiangsu Cowin Biotech Co., Ltd., Taizhou, China), and the ITS, *rpb1*, and *tef-1α* gene regions were amplified by polymerase chain reaction (PCR) using the primers listed in Table 1. The PCR conditions for ITS were as follows: initial denaturation at 95 °C for 3 min; 30 cycles of 95 °C for 30 s, 54 °C for 30 s, and 72 °C for 1 min; and a final extension at 72 °C for 10 min [20]. For *rpb1* and *tef-1α*, they were as follows: initial denaturation at 94 °C for 60 s; 10 cycles of 94 °C for 35 s, 53 °C for 45 s, and 72 °C for 45 s; 25 cycles of 94 °C for 35 s, 56 °C for 45 s, and 72 °C for 45 s; and a final extension at 72 °C for 10 min [6,21].

### 2.3. Phylogenetic Tree Construction

Newly obtained sequences were submitted to NCBI GenBank. To construct the phylogenetic tree, sequences with high similarity (BLAST similarity > 94%) from taxa phylogenetically and morphologically close to *Prunulus* were downloaded from GenBank (https://www.ncbi.nlm.nih.gov/genbank/, accessed on 25 November 2025). *Mycena meliigena* (Berk. & Cooke) Sacc. and *M. metata* (Fr.) P. Kumm. were selected as the outgroup. All sequence information is listed in Table 2. The ITS, *rpb1*, and *tef-1α* sequences were aligned separately in BioEdit v7.2.5, with subsequent manual adjustments [23], and then concatenated using PhyloSuite v1.2.3 [24]. Phylogenetic trees were constructed using maximum likelihood (ML) and Bayesian inference (BI) methods. The ML tree was constructed in MEGA 11 under the best-fit HKY + G model. For the BI analysis [25], the best-fit HKY + F + I + G4 model was selected using PhyloSuite v1.2.3 [24]. The phylogenetic trees were visualized using FigTree v1.4.4 and finalized with Adobe Photoshop 2024.

## 3. Results

### 3.1. Phylogenetic Analysis

**Figure 1:**
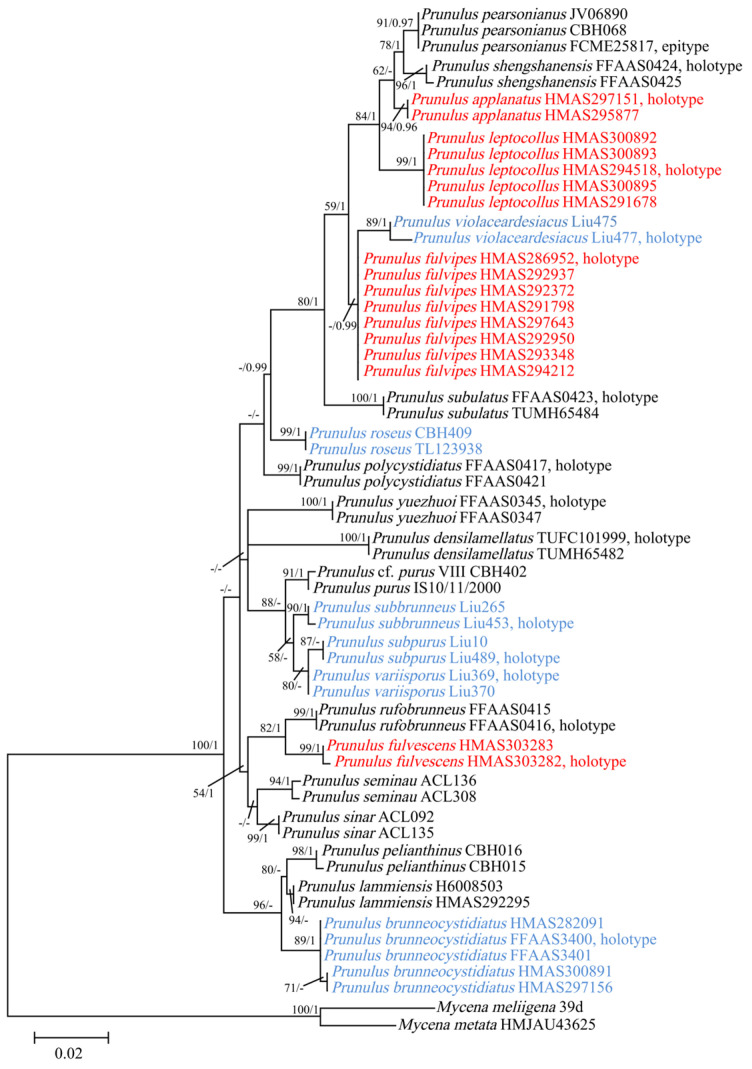
ML phylogram based on the ITS + *rpb1* + *tef-1α* dataset for *Prunulus*. ML bootstrap values (ML) ≥ 50% and Bayesian posterior probabilities (BPP) ≥ 0.95 are shown on the branches (ML/BPP). New species are indicated in red text, and new combinations are in blue text.

Phylogenetic analysis was performed using a combined dataset comprising ITS, *rpb1*, and *tef-1α* sequences, with 138 sequences (59 for ITS, 39 for *rpb1*, and 40 for *tef-1α*) from 59 specimens representing 24 species. The phylogenetic topologies generated by the ML and BI analyses were highly congruent, and the ML tree was selected for presentation. The samples of the four new species described below, namely, *P. applanatus*, *P. fulvescens*, *P. fulvipes*, and *P. leptocollus*, formed four well-supported, distinct lineages (Figure 1). *Prunulus applanatus* (94/0.96) was recovered as a sister to the clade containing *P. pearsonianus* (Dennis ex Singer) Kun L. Yang, Jia Y. Lin & Zhu L. Yang and *P. shengshanensis* (Z.W. Liu, Y.P. Ge & Q. Na) Kun L. Yang, Jia Y. Lin & Zhu L. Yang. These three species further formed a clade (84/1) as a sister to another new species, *P. leptocollus* (99/1). *Prunulus fulvipes* formed a clade with *P. violaceardesiacus* (Shun Liu & Biao Zhu) Rui Wang bis, Ke Wang, H.F. Liu, Di Liu & T.Z. Wei, with strong BI support (BPP = 0.99), whereas the ML bootstrap support was very low, potentially due to the incomplete ITS sequence of *P. violaceardesiacus* (483 bp). The ITS sequence of *P. fulvipes* shared 98% identity with that of *P. violaceardesiacus* (PP037953), while the *tef-1α* sequences of each shared 97% identity (PP034088). Combined with their distinct morphological characteristics, these data clearly distinguish the two species. In a separate part of the tree, *P. fulvescens* (99/1) and *P. rufobrunneus* (Z.W. Liu, Y.P. Ge & Q. Na) Kun L. Yang, Jia Y. Lin & Zhu L. Yang formed a sister clade (82/1).

According to the results of the phylogenetic analysis, *M. rosea* Gramberg and five recently proposed *Mycena* species, such as *M. brunneocystidiata* Jing W. Guo, Ze W. Liu, Y.P. Ge & Q. Na, *M. subbrunnea* Shun Liu & Biao Zhu, *M. subpura* Shun Liu & Biao Zhu, *M. variispora* Shun Liu & Biao Zhu, and *M. violaceardesiaca* Shun Liu & Biao Zhu, can be integrated into the group of *Prunulus* species.

### 3.2. Taxonomy

#### 3.2.1. Species Reassociation

Morphologically, compared to other taxa within *Mycena*, *Prunulus* species share similar purplish, brownish or pinkish pilei, elliptical to cylindrical basidiospores, smooth pileipelles, and fusiform to clavate cystida [15,17,26]. Phylogenetic analysis of traditional *Mycena* species supports the transfer of *Mycena* sect. *Calodontes* to *Prunulus*, with *P. pelianthinus* (*M. pearsoniana*) serving as the type species [2]. According to the phylogenetic tree we constructed from three genes (Figure 1), *M. rosea* and five new *Mycena* species form a strongly supported clade with *Prunulus* species. Therefore, we propose to transfer the following species to *Prunulus*:

*Prunulus brunneocystidiatus* (Jing W. Guo, Ze W. Liu, Y.P. Ge & Q. Na) Rui Wang bis, Ke Wang, H.F. Liu, Di Liu & T.Z. Wei, comb. nov.

Registration identifier: FN 573192.

Basionym: *Mycena brunneocystidiata* Jing W. Guo, Ze W. Liu, Y.P. Ge & Q. Na, in Guo, Liu, Zeng, Ge & Na, *MycoKeys* 125: 57 (2025).

Notes: *Prunulus brunneocystidiatus* has been described in mixed coniferous–broadleaf forests of *Larix*, *Pinus*, and *Quercus* in Jilin Province, characterized by brown pilei, smooth, narrowly ellipsoid basidiospores, fusiform cheilocystidia, and pleurocystidia with brownish contents. This new combination was proposed based on a phylogenetic analysis of sequence data from its holotype, FFAAS3400 (from China, with ITS sequence PV939239) [17]. This study extends the recorded distribution of this species to Shanxi Province, based on specimen HMAS300891.

*Prunulus roseus* (Gramberg) Rui Wang bis, Ke Wang, H.F. Liu, Di Liu & T.Z. Wei, comb. nov.

Registration identifier: FN 573193.

Basionym: *Mycena rosea* Gramberg, *Pilze Heimat* 1: 36 (1912).

Notes: *Prunulus roseus* has been described in mixed coniferous–broadleaf forests of *Fagus, Quercus*, and *Pinus*, and is widely distributed across Europe, North America, Asia, and Africa. In China, it is distributed across Inner Mongolia, Jilin, Hebei, Jiangxi, Guizhou, and Yunnan. The species is characterized by its pale pink to lilaceous pink pileus, pip-shaped basidiospores, and fusiform to clavate cheilocystidia [26,27]. This new combination was proposed based on a phylogenetic analysis of sequence data from specimen CBH409 (from Germany, with ITS sequence FN394551) [5].

*Prunulus subbrunneus* (Shun Liu & Biao Zhu) Rui Wang bis, Ke Wang, H.F. Liu, Di Liu & T.Z. Wei, comb. nov.

Registration identifier: FN 573194.

Basionym: *Mycena subbrunnea* Shun Liu & Biao Zhu, in Liu, Cui & Zhu, *Mycology* 16(2): 720 (2024).

Notes: *Prunulus subbrunneus* has been described in mixed coniferous–broadleaf forests of *Betula*, *Larix*, and *Pinus* in Hebei Province, and is characterized by a pinkish buff to buff-yellow pileus, cylindrical basidiospores, and clavate to fusiform cheilocystidia. This new combination was proposed based on a phylogenetic analysis of sequence data from its holotype, Liu453 (from China, with ITS sequence PP037951) [15].

*Prunulus subpurus* (Shun Liu & Biao Zhu) Rui Wang bis, Ke Wang, H.F. Liu, Di Liu & T.Z. Wei, comb. nov.

Registration identifier: FN 573195.

Basionym: *Mycena subpura* Shun Liu & Biao Zhu, in Liu, Cui & Zhu, *Mycology* 16(2): 724 (2024).

Notes: *Prunulus subpurus* has been described in mixed forests of *Betula*, *Larix,* and *Picea* in Hebei Province, and is characterized by a glabrous, pale vinaceous pileus, a purple-brown stipe, elongated ellipsoid basidiospores, and clavate cheilocystidia. This new combination was proposed based on a phylogenetic analysis of sequence data from its holotype, Liu489 (from China, with ITS sequence PP037954) [15].

*Prunulus variisporus* (Shun Liu & Biao Zhu) Rui Wang bis, Ke Wang, H.F. Liu, Di Liu & T.Z. Wei, comb. nov.

Registration identifier: FN 573196.

Basionym: *Mycena variispora* Shun Liu & Biao Zhu, in Liu, Cui & Zhu, *Mycology* 16(2): 724 (2024).

Notes: *Prunulus variisporus* has been described in mixed forests of *Betula* and *Larix* in Hebei Province, and is characterized by a cream to lilac pileus, a grayish violet stipe, cylindrical basidiospores, and clavate cheilocystidia. This new combination was proposed based on a phylogenetic analysis of sequence data from its holotype, Liu369 (from China, with ITS sequence PP037949) [15].

*Prunulus violaceardesiacus* (Shun Liu & Biao Zhu) Rui Wang bis, Ke Wang, H.F. Liu, Di Liu & T.Z. Wei, comb. nov.

Registration identifier: FN 573197.

Basionym: *Mycena violaceardesiaca* Shun Liu & Biao Zhu [as ‘violocea-ardesiaca’], in Liu, Cui & Zhu, *Mycology* 16(2): 725 (2024).

Notes: *Prunulus violaceardesiacus* has been described in broad-leaved forests of *Betula* in Hebei Province, and is characterized by a pale vinaceous to lilac pileus, a grayish violet stipe, ellipsoid basidiospores, and clavate cheilocystidia. This new combination was proposed based on a phylogenetic analysis of sequence data from its holotype, Liu477 (from China, with ITS sequence PP037953) [15].

#### 3.2.2. *Prunulus applanatus* Rui Wang bis, Ke Wang, X.D. Yu, H.F. Liu, Di Liu & T.Z. Wei, sp. nov. Figure 2 and Figure 3

Registration identifier: FN 573188.

Etymology: The epithet *applanatus* refers to the pileus gradually expanding from a hemispherical to flattened shape.

Holotype: China. Liaoning Province, Dandong City, Kuandian Manchu Autonomous County, Tianqiaogou Forest Park, alt. 600 m, 14 August 2021, Fei Xu 22, HMAS297151.

Diagnosis: Basidiocarps small-sized. Pileus applanate, distinctly striate at margin, pale pink to pale grey-brown. Lamellae moderately crowded, pale pinkish to whitish. Stipe cylindrical, central, pale brown to grayish brown. Basidiospores weakly amyloid, 6–8 × 3.5–4 μm, narrowly ellipsoid. Cheilocystidia subcylindrical to sublageniform.

**Figure 2:**
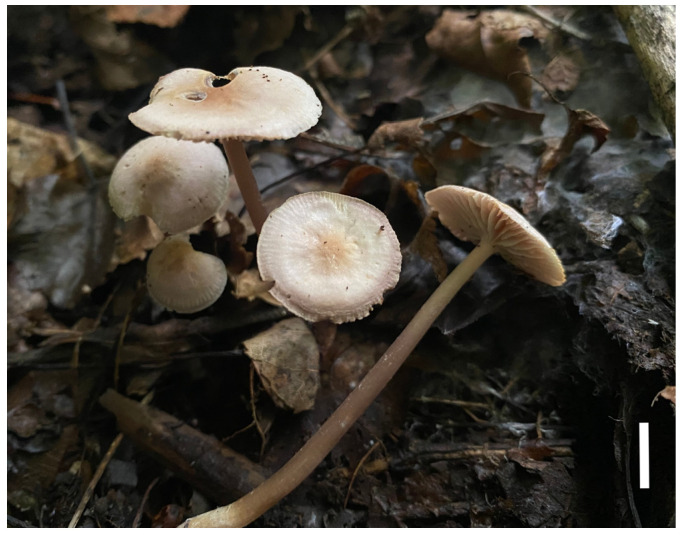
Basidiomata of *Prunulus applanatus* sp. nov. (HMAS297151, holotype). Scale bar: 1 cm.

Description: Pileus 15–35 mm in diam., initially hemispherical, expanding to plano-convex to applanate when mature, sometimes slightly umbonate at center; surface glabrous, hygrophanous when moist, distinctly striate at margin; pale pinkish to pale grey-brown with slightly lilac tint, brown at the center, pale lilac at margin; texture fragile and thin. Lamellae adnate, moderately crowded, with lamellulae, 2–4 mm wide; pale pinkish to whitish with lilac tint. Stipe 30–75 × 2–4 mm, central, cylindrical, slightly enlarged at base, fragile; surface longitudinally striate, glabrous or occasionally with whitish fibrils; light to pale brown or grayish brown, with whitish fibrils at base. Odor indistinct. Taste mild.

**Figure 3:**
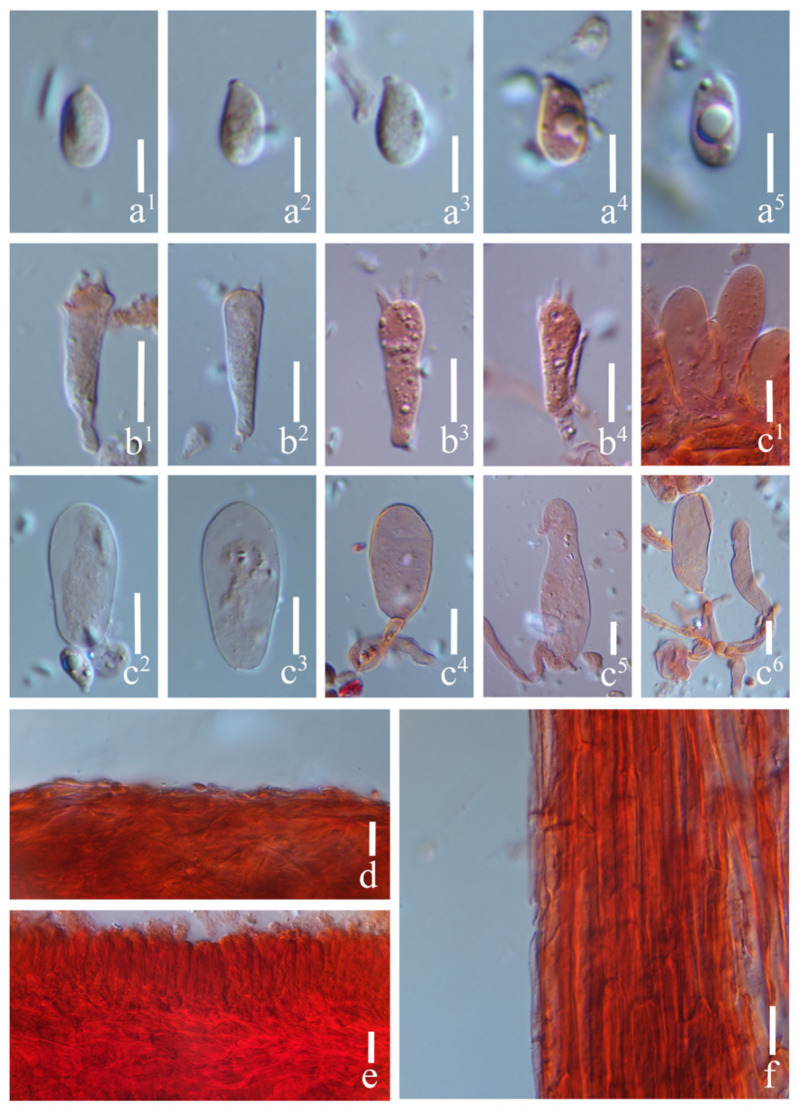
Microstructures of *Prunulus applanatus* (HMAS297151, holotype). (**a^1^**–**a^5^**) Basidiospores; (**b^1^**–**b^4^**) basidia; (**c^1^**–**c^6^**) cheilocystidia; (**d**) pileipellis; (**e**) lamellae trama; (**f**) stipitipellis. Scale bars: (**a^1^**–**a^5^**) = 5 μm; (**b^1^**–**f**) = 10 μm.

Basidiospores (5.5–) 6–8 (–8.5) × (3–) 3.5–4 (–4.5) μm, Q = 1.50–2.31, Qav = 1.80 ± 0.20; narrowly ellipsoid to cylindrical, hyaline, smooth, thin-walled, sometimes containing oil droplets, weakly amyloid. Basidia 15–27 × 4–7.5 μm, narrowly clavate, subhyaline, smooth, thin-walled, 4-spored, sterigmata up to 6.0 μm long. Cheilocystidia 17–70 × 11–15 μm, clavate, obovate, subcylindrical to sublageniform, apically obtuse, hyaline, smooth, thin-walled. Pleurocystidia absent. Pileipellis a cutis of parallel hyphae, 2.5–5 μm in diam., hyaline, smooth, thin-walled. Lamellae trama regular, of subcylindrical hyphae, 4–12 μm in diam., hyaline, thin-walled. Stipitipellis a cutis of narrow hyphae, 3–6 μm in diam., hyaline, smooth, thin-walled. Clamp connections present in all tissues, but rarely observed in the context.

Habitat and distribution: Gregarious on decaying wood and humus layer in deciduous broad-leaved forests, mainly under trees of *Quercus* and *Juglans*. Known in Liaoning and Anhui Provinces in China.

Materials examined: China. Anhui Province, Lu’an City, Jinzhai County, Tiantangzhai Scenic Area, 11 October 2020, Yao-Bin Guo 1539, HMAS295877.

Notes: *Prunulus applanatus* is characterized by a pale and applanate pileus with slightly lilac tint, and distinctly striate at pileus margin. Phylogenetically and morphologically, *P. applanatus* is close to *P. pearsonianus* [26] and *P. shengshanensis* [6]. Both of the latter two species have lilac to violaceus-tinged basidiomata, striate pileus, and clavate to sublageniform cheilocystidia. However, compared to *P. applanatus*, the two related species have obviously darker pilei. In contrast with the new species, *P. pearsonianus* has a distinct violaceus pileus [26,28], and the pileus of *P. shengshanensis* is violaceous-brown [6].

#### 3.2.3. *Prunulus fulvescens* Rui Wang bis, Ke Wang, H.F. Liu, Di Liu & T.Z. Wei, sp. nov. Figure 4 and Figure 5

Registration identifier: FN 573189.

Etymology: The epithet *fulvescens* refers to the color change of the pileus from dark brown to yellowish-brown.

Holotype: China. Sichuan Province, Garzê Tibetan Autonomous Prefecture, Jiulong County, alt. 3575 m, 16 July 2024, Rui Wang 90, HMAS303282.

Diagnosis: Basidiocarps medium-sized. Pileus with distinct blackish-brown streaks, black-brown to reddish-brown when young, and yellowish-brown, light grayish-purple when mature. Lamellae subdistant, grayish-purple, yellowish-brown when fully mature. Stipe cylindrical, glabrescent, dark purple to purplish-brown and base with sparse whitish fibrils. Basidiospores 6.8–8.7 × 3.7–5.5 μm, narrowly ellipsoid. Cheilocystidia obovate to elongate ovoid.

Description: Pileus 25–50 mm in diam., hemispherical when young, becoming convex to plano-convex when mature, sometimes umbilicate at the center; surface glabrous, hygrophanous when moist, margin wavy and cracked with age, with distinct blackish-brown streaks; firstly black-brown, reddish-brown to purplish-brown, with grayish-brown, gradually paling to pale reddish-brown to yellowish-brown, with purplish tint when mature; texture fragile and thin. Lamellae slightly decurrent to adnate, subdistant, with lamellulae, 2–4 mm wide, grayish-purple, grayish-white to yellowish-brown. Stipe 20–35 × 3–6 mm, cylindrical, central, fragile; surface glabrescent, dark purple to purplish-brown, with a slightly swollen base sparsely covered with whitish fibrils. Odor indistinct. Taste mild.

**Figure 4:**
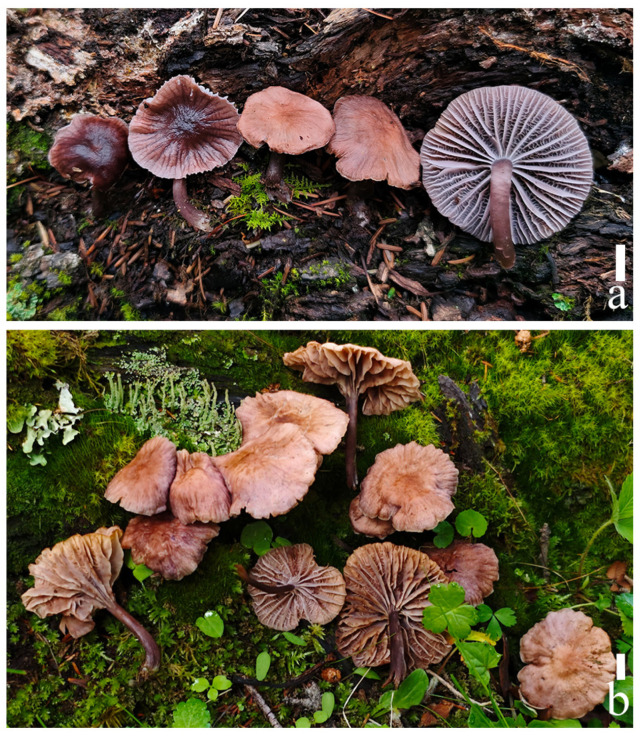
Basidiomata of *Prunulus fulvescens* sp. nov. (**a**) HMAS303282 (holotype); (**b**) HMAS303283. Scale bar: 1 cm.

**Figure 5:**
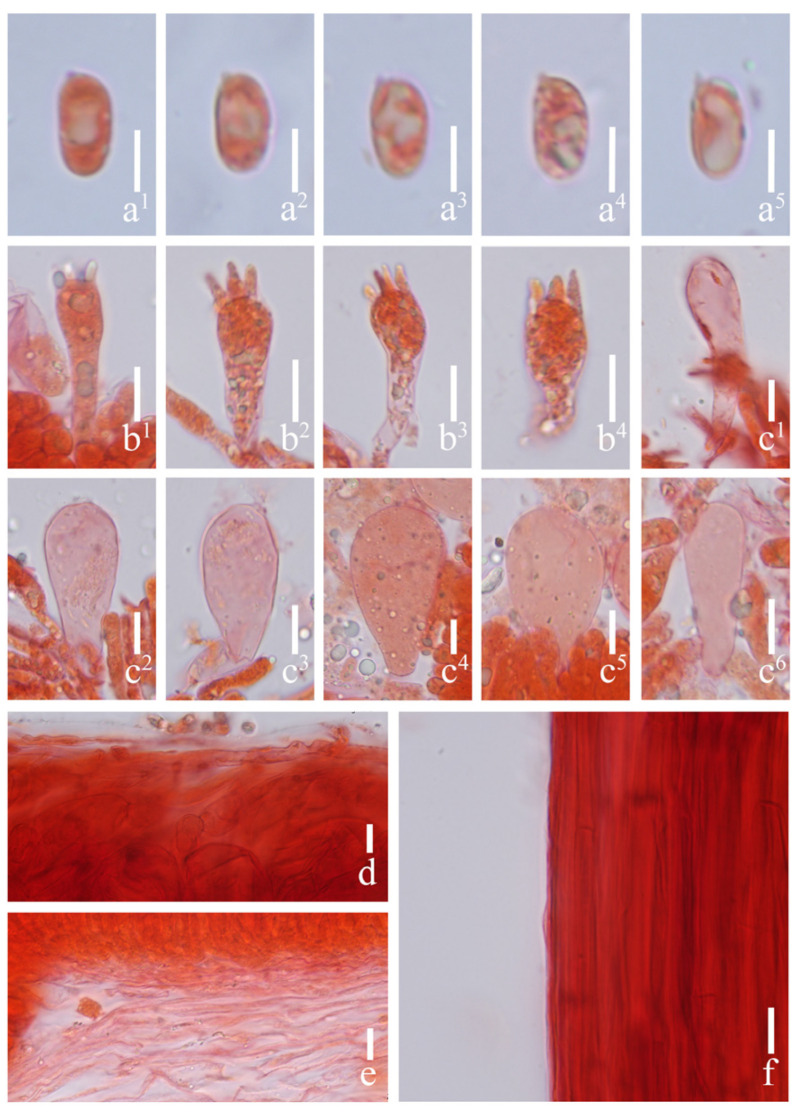
Microstructures of *Prunulus fulvescens*. (HMAS303282, holotype). (**a^1^**–**a^5^**) Basidiospores; (**b^1^**–**b^4^**) basidia; (**c^1^**–**c^6^**) cheilocystidia; (**d**) pileipellis; (**e**) lamellae trama; (**f**) stipitipellis. Scale bars: (**a^1^**–**a^5^**) = 5 μm; (**b^1^**–**f**) = 10 μm.

Basidiospores (6.5–) 6.8–8.7 (–9.0) × (3.2–) 3.7–5.5 (–6.0) μm, Q = 1.4–2.2, Qav = 1.79 ± 0.19; cylindrical to narrowly ellipsoid, hyaline, smooth, thin-walled, containing oil droplets, amyloid. Basidia 19–32 × 5–9 μm, clavate, subhyaline, smooth, thin-walled, 4-spored, sterigmata up to 6.5 μm long. Cheilocystidia 20–48 × 9–26.5 μm, clavate, obovate to elongate ovoid, apically obtuse, smooth, hyaline, thin-walled. Pleurocystidia absent. Pileipellis a cutis of parallel hyphae, 2.5–6.5 μm in diam., smooth, hyaline, thin-walled. Lamellae trama regular, of cylindrical hyphae, 4–17 μm in diam., hyaline, slightly thick-walled. Stipitipellis a cutis of cylindrical hyphae, 3–7.5 μm in diam., smooth, hyaline, thin-walled. Clamp connections present in all tissues, but rarely observed in the context.

Habitat and distribution: Gregarious or scattered on moss-covered wood in subalpine mixed coniferous–broadleaf forests, mainly under trees of *Abies* and *Pinus*. Known in Sichuan Province in China.

Materials examined: China. Sichuan Province, Garzê Tibetan Autonomous Prefecture, Jiulong County, 16 July 2024, Peng Hong 920, HMAS303283.

Notes: *Prunulus fulvescens* is characterized by its dark brown pileus when young, which becomes yellowish-brown when mature. According to results of phylogenetic analysis, *P. fulvescens* is close to *P. rufobrunneus*, which has brown pileus and purple-to-violet-tinged stipe [6]. However, *P. rufobrunneus* does not have purplish pilei and lamellae [6]. *Prunulus seminau* and *P. sinar* are also closely related to the new species, but neither species have a purplish tint at any life stage [10].

#### 3.2.4. *Prunulus fulvipes* Rui Wang bis, Ke Wang, H.F. Liu, Di Liu & T.Z. Wei, sp. nov. Figure 6 and Figure 7

Registration identifier: FN 573190.

Etymology: The epithet *fulvipes* refers to the persistent yellowish-brown color of the stipe base.

Holotype: China. Hebei Province, Baoding City, Fuping County, Tianshengqiao National Geological Park, alt. 1400 m, 23 August 2020, Yao-Bin Guo 1254, HMAS286952.

Diagnosis: Basidiocarps medium-sized. Pileus surface glabrous, translucent-striate at margin, brownish-white, then pale brown at the center. Lamellae crowded, light yellowish white to pale lilac. Stipe longer, cylindrical, slightly hygrophanous, pale pinkish to lilac white, and the base is yellowish-brown. Basidiospores ellipsoid, 5.6–7.6 × 3.8–5.5 μm. Cheilocystidia cylindrical to fusiform.

**Figure 6:**
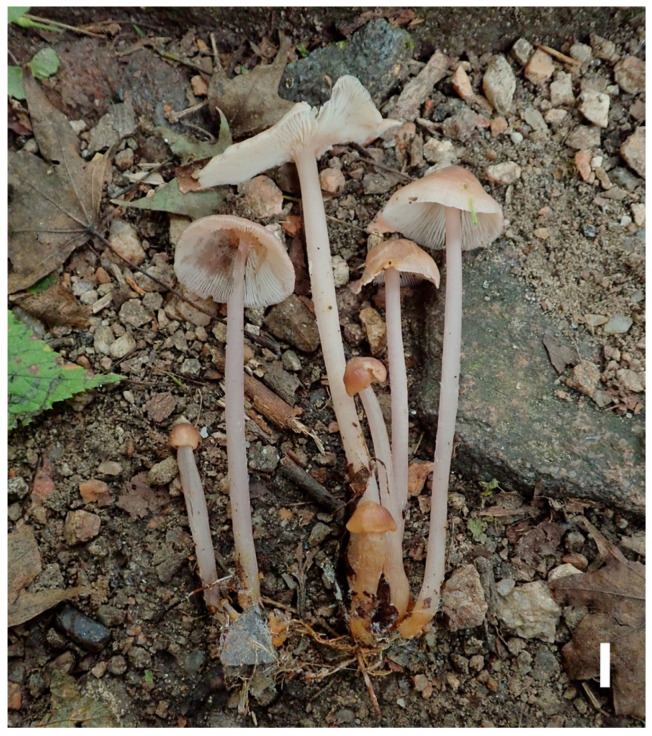
Basidiomata of *Prunulus fulvipes* sp. nov. (HMAS286952, holotype). Scale bar: 1 cm.

Description: Pileus 21–50 mm in diam., hemispherical to campanulate at first, becoming convex to applanate when mature, sometimes umbonate at center; surface glabrous, hygrophanous when moist, slightly radically translucent-striate at margin; light brown, yellowish brown to light reddish brown when young, pale brown at center, and brownish white to cream-white elsewhere when mature, texture fragile and thin. Lamellae sinuate to slightly decurrent, crowded, with lamellulae, 2–3 mm wide; light yellowish white to pale lilac. Stipe 60–140 × 3–5 mm, cylindrical, central, fragile; surface longitudinally striate, slightly hygrophanous when moist, covered with sparse whitish fibrils when young; pale yellowish-brown to light brownish pink when young, fading to pale pinkish to lilac white when mature, covered whitish fibrils at base. Odor indistinct. Taste mild.

Basidiospores (5.4–) 5.6–7.6 (–7.9) × (3.5–) 3.8–5.5 (–5.7) μm, Q = 1.14–1.73, Qav = 1.42 ± 0.15; ellipsoid, hyaline to subhyaline, smooth, thin-walled, sometimes containing oil droplets, amyloid. Basidia 12–22 × 5–9.5 μm, clavate, smooth, 2- to 4-spored, sterigmata up to 4 μm long. Cheilocystidia 29–57 × 4–17 μm, cylindrical to fusiform, tapering to a constricted apex, smooth, hyaline, thin-walled, apically narrowed to a generally acute apex. Pleurocystidia absent. Pileipellis a cutis of narrow hyphae, 2–5.5 μm in diam., hyaline, smooth. Lamellae trama subregular, of cylindrical hyphae 2–6.5 μm in diam., hyaline, smooth, thin-walled. Stipitipellis a cutis of hyphae, 4–6.5 μm in diam., hyaline, smooth. Clamp connections present in all tissues, but rarely observed in the context.

**Figure 7:**
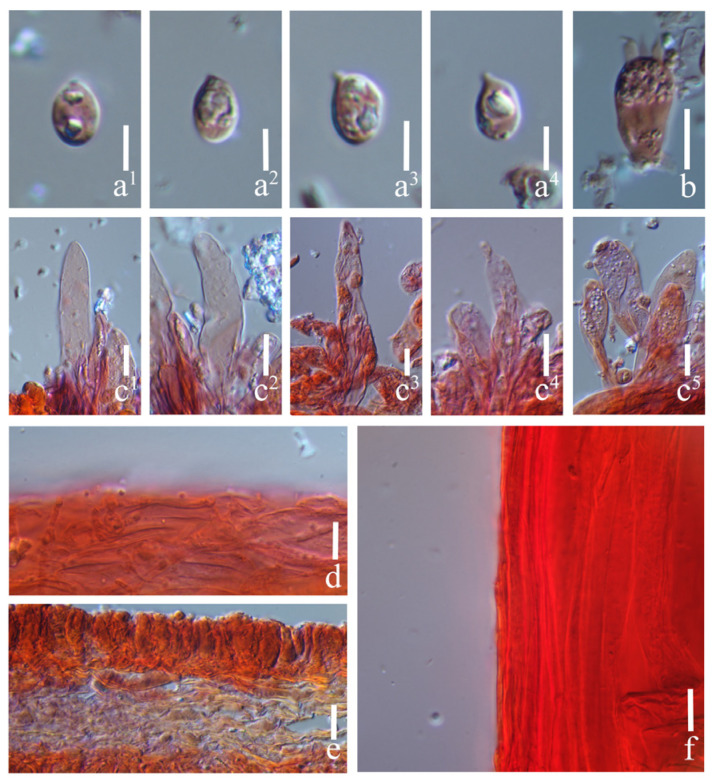
Microstructures of *Prunulus fulvipes*. (HMAS286952, holotype). (**a^1^**–**a^4^**) Basidiospores; (**b**) basidia; (**c^1^**–**c^5^**) cheilocystidia; (**d**) pileipellis; (**e**) lamellae trama; (**f**) stipitipellis. Scale bars: (**a^1^**–**a^4^**) = 5 μm; (**b**–**f**) = 10 μm.

Habitat and distribution: Gregarious on humus-rich sandy soil in mixed coniferous–broadleaf forests, mainly under trees of *Acer*, *Betula*, and *Pinus.* Known in Hebei Province and Beijing City in China.

Materials examined: China. Beijing City, Fangshan District, Baihuashan National Nature Reserve, 25 August 2020, Tie-Zheng Wei B-4, HMAS292372. Hebei Province, Baoding City, Fuping County, Tianshengqiao National Geological Park, 2 August 2020, Yao-Bin Guo 799, HMAS297643; *ibid*, Xia-Nan Shan 79, HMAS292950; Shijiazhuang City, Pingshan County, Tuoliang National Nature Reserve, 16 August 2019, Yao-Bin Guo 49, HMAS291798; *ibid*, 22 August 2020, Yao-Bin Guo 1197, HMAS293348; *ibid*, Xu Zhang 165, HMAS294212; *ibid*, Xia-Nan Shan 64, HMAS292937.

Notes: *Prunulus fulvipes* is characterized by a pileus with a brown center and a lighter brownish-white margin, longer stipe with a yellowish-brown base. Phylogenetically and morphologically, *P. fulvipes* is close to *P. violaceardesiacus*, and the latter also has a vinaceous to lilac tint [15]. However, it differs from the vinaceous basidiomata of *P. violaceardesiacus*, with the new species having a distinctly more brown tint.

#### 3.2.5. *Prunulus leptocollus* Rui Wang bis, Ke Wang, H.F. Liu, Di Liu & T.Z. Wei, sp. nov. Figure 8 and Figure 9

Registration identifier: FN 573191.

Etymology: The epithet *leptocollus* refers to the cheilocystidia with a slightly narrowed neck.

Holotype: China. Hebei Province, Baoding City, Laishui County, Yesanpo Scenic Area, alt. 1274 m, 28 August 2020, Tong-Kai Zong 335, HMAS294518.

Diagnosis: Basidiocarps small-sized, pileus applanate, surface glabrous, translucent-striate, pale lilac to pale brown with lilac tint. Lamellae crowded, lilac white to lilac creamy. Stipe cylindrical, surface glabrescent, longitudinally striate, lilac to pale purplish-pink. Basidiospores ellipsoid, 6.5–8.4 × 4–5.5 μm. Cheilocystidia clavate, with a slightly narrowed neck. Caulocystidia subcylindrical.

**Figure 8:**
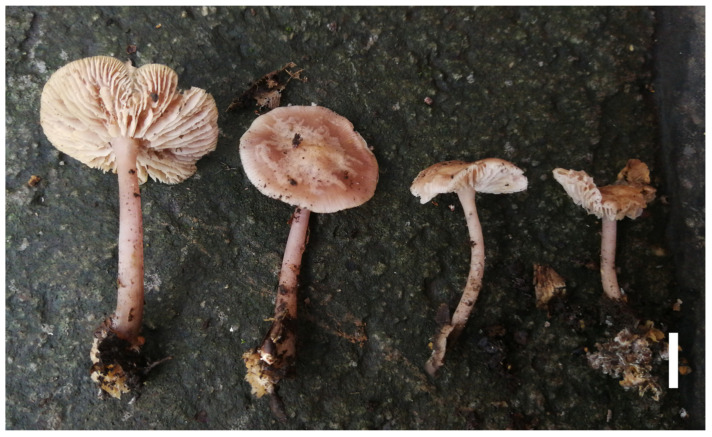
Basidiomata of *Prunulus leptocollus* sp. nov. (HMAS294518, holotype). Scale bar: 1 cm.

Description: Pileus 25–35 mm in diam., hemispherical to convex when young, applanate with shallow umbonate center when mature, margin sometimes wavy or uplifted, rarely splitting; surface glabrous, sulcate, radically translucent-striate, hygrophanous when moist; pale lilac to pale brown with lilac tints, creamy to whitish at the margin; texture fragile and thin. Lamellae sinuate, crowded, with lamellulae, 2–4 mm wide; lilac white to lilac creamy. Stipe 25–50 × 2–5 mm, central, cylindrical, slightly enlarged at base, fragile; surface glabrescent, longitudinally striate; pale lilac to pale purplish-pink, base slightly darker and covered with sparse whitish hyphae. Odor indistinct. Taste mild.

Basidiospores (6.0–) 6.5–8.4 (–9.0) × (3.5–) 4–5.5 (–6) μm, Q = 1.31–2.01, Qav = 1.58 ± 0.17; ellipsoid, hyaline, smooth, thin-walled, sometimes containing oil droplets, amyloid. Basidia 15–30 × 4.5–9.5 μm, clavate, hyaline, smooth, 4-spored, sterigmata up to 4.5 μm long. Cheilocystidia 18–50 × 5–13 μm, cylindrical to clavate, mostly tapering at upper part, sometimes slightly expanded at apex, hyaline, smooth, thin-walled. Pleurocystidia absent. Pileipellis a cutis of hyphae 2.5–6 μm in diam., hyaline, smooth. Lamellae trama regular, of cylindrical hyphae, 2–11 μm in diam., hyaline, smooth, thin-walled. Stipitipellis a cutis of cylindrical hyphae, 3–7 μm in diam., hyaline, smooth. Caulocystidia 12–54 × 3–10 μm, subcylindrical, tapering toward apex, hyaline, smooth, thin-walled. Clamp connections present in all tissues, but rarely observed in the context.

Habitat and distribution: Scattered and gregarious on rotten wood and humus in mixed coniferous–broadleaf forests, mainly under trees of *Populus*, *Betula*, and *Pinus*. Known in Hebei Province and Beijing City in China.

Materials examined: China. Beijing City, Yanqing District, Songshan National Nature Reserve, 23 August 2022, Jing Yang 720, HMAS300892; *ibid*, 9 September 2023, Tie-Zheng Wei 9917, HMAS300895. Hebei Province, Chengde City, Weichang County, Saihanba National Forest Park, 21 August 2021, Shi-Yi Zhao 20210641, HMAS300893; Shijiazhuang City, Pingshan County, Tuoliang National Nature Reserve, 15 August 2019, Yao-Bin Guo 10, HMAS291678.

Notes: *Prunulus leptocollus* is characterized by its pale lilac basidiomata, and its cheilocystidia have narrowed necks and obtuse apexes. According to results of the phylogenetic analysis (Figure 1.), *P. leptocollus* forms a separate branch with three lilac species: *P. applanatus*, *P. pearsonianus,* and *P. shengshanensis*. However, the three related species do not have cheilocystidia with narrowed necks [6,26]. *Prunulus leptocollus* is markedly darker than *P. applanatus*, and its basidiospores (up to 6 μm wide) are wider than those of *P. applanatus* (up to 4.5 μm), *P. pearsonianus* (up to 5 μm) [26], and *P. shengshanensis* (up to 4.7 μm) [6].

**Figure 9:**
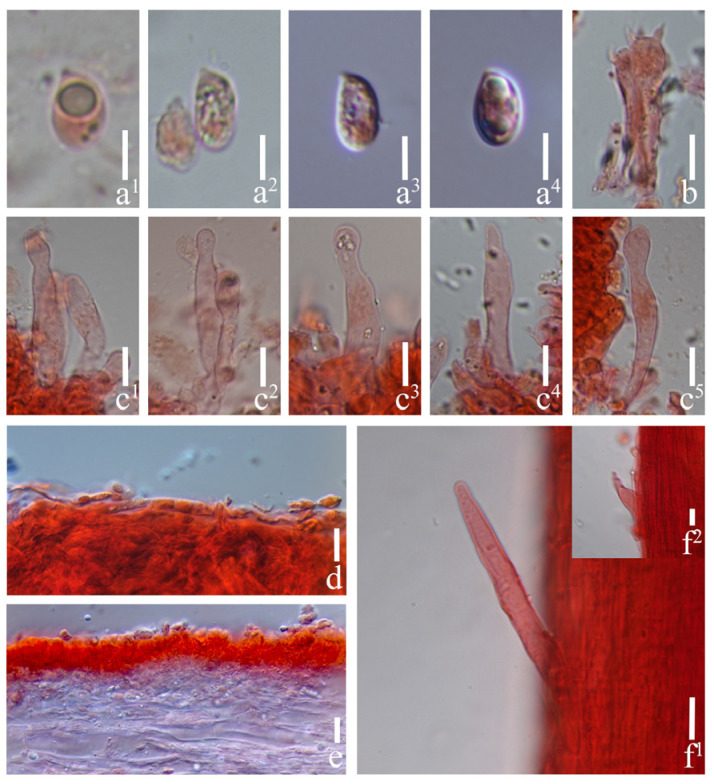
Microstructures of *Prunulus leptocollus*. (HMAS294518, holotype). (**a^1^**–**a^4^**) Basidiospores; (**b**) basidia; (**c^1^**–**c^5^**) cheilocystidia; (**d**) pileipellis; (**e**) lamellae trama; (**f^1^**,**f^2^**) stipitipellis. Scale bars: (**a^1^**–**a^4^**) = 5 μm; (**b**–**f^2^**) = 10 μm.

## 4. Discussion

Among the four new species described in this study, caulocystidia were only observed in *P. leptocollus*, and the basidiospores of *P. applanatus* were weakly amyloid. Notably, *P. fulvescens* was collected from mixed coniferous–broadleaf forests in a subalpine region (alt: 3575 m), whereas the three new species, like most known members of *Prunulus*, occur predominantly in low-altitude areas. This finding represents a new recording of the genus existing at a high altitude. *Prunulus* species generally show a preference for cool–temperate coniferous or mixed coniferous–broadleaf forests and are less reported in subtropical and tropical regions in China [6,13,17,27]. This ecological tendency helps explain the occurrence of *P. fulvescens* in high-altitude habitats in southwest China.

*Mycena* is a species-rich genus with complex interspecific relationships. Maas Geesteranus established an infrageneric taxonomic system comprising 44 sections, primarily based on the color of the pileus and stipe [8,9]. However, molecular phylogenetic studies consistently indicate that *Mycena* is a polyphyletic group [27,29,30]. Yang et al. (2025) reassigned the species formerly placed in *Mycena* sect. *Calodontes* to the genus *Prunulus* [2]. Morphologically, *M. rosea* and several recently described *Mycena* spp. from China share similar purplish to pinkish pilei, fusiform to clavate cheilocystidia, and a smooth pileipellis with *Prunulus*. Their relationship is also strongly supported by molecular evidence, and consequently, we accommodate them within *Prunulus*.

*Prunulus* exhibits diverse pileus colors and occupies a wide range of habitats, occurring mainly on dead wood, in plant litter, and in sandy soil within coniferous and broad-leaved forests across the Northern Hemisphere [10,11,17,21,26,27]. Key morphological characteristics for species delineation in *Prunulus* include the pileus color and morphology of basidiospores and cystidia [6,11,17]. For example, *P. subpurus* and *P. violaceardesiacus* share a similar pileus color and cystidia, but the basidiospores of *P. violaceardesiacus* are broader (5.2 μm) than those of the former [15]. Additionally, *P. leptocollus* and *P. shengshanensis* [6] can be distinguished by the shape of their cheilocystidia. Complexes of *P. pearsonianus* and *P. purus* comprising phylogenetically distinct lineages indicate many cryptic species [21,28]. Recently, two new species, *P. variisporus* and *P. subpurus*, have been described as being part of the *P. purus* complex, from China [15], and the results of multigene phylogenetic analyses and microscopic characteristics have proven useful for delimiting these undescribed taxa.

## 5. Conclusions

During our extensive surveys on macrofungi in China, four new species were described based on morphological and multigene phylogenetic evidence, namely, *P. applanatus*, *P. fulvescens*, *P. fulvipes*, and *P. leptocollus*. Furthermore, the incorporation of six species previously classified in *Mycena* sect. *Calodontes* into the genus *Prunulus* is proposed. These taxonomic revisions are intended to contribute to a clearer taxonomy of *Prunulus* species.

**Key for related *Prunulus* species in this study:**
1. Basidiomata lilac-, vinaceous-, purplish- or violet-tinged21. Basidiomata without lilac, vinaceous, purplish, or violet tint162. Pileus without lilac, vinaceous, purplish, or violet tint32. Pileus lilac-, vinaceous-, purplish- or violet-tinged43. Pileus brownish-white; lamellae lilac-tinged; stipe pinkish to lilac white; basidiospores 5.6–7.6 × 3.8–5.5 μm
*P. fulvipes*
3. Pileus dark brown at center, elsewhere reddish-brown to greyish-brown; lamellae white; stipe greyish magenta, dull violet to dark purple; basidiospores 7.1–9.6 × 3.8–5.0 μm
*P. rufobrunneus*
4. Pileus with distinct blackish-brown streaks; lamellae subdistant, grayish-purple when young; stipe dark purple to purplish brown; basidiospores 6.8–8.7 × 3.7–5.5 μm
*P. fulvescens*
4. Pileus without distinct blackish streaks55. Lamellae edge darker, dark-violet-tinged65. Lamellae edge concolorous with lamellae76. Pileus sordid lilac; stipe pale sordid lilac; basidiospores 6–9 × 3.5–4.5 μm
*P. lammiensis*
6. Pileus pale lilac brown to pale purplish brown; stipe pale with lilac tint
*P. pelianthinus*
7. Lamellae whitish when mature87. Lamellae not whitish when mature98. Pileus brown to violet-brown; stipe violet-tinged; chelocystiia clavate with slightly inflate apex; basidiospores 6–8.7 × 3.4–4.7 μm
*P. shengshanensis*
8. Pileus greyish-rose, with brownish-orange umbo; stipe pubescent; basidiospores 6.4–8.8 × 3.2–4.6 μm
*P. polycystidiatus*
9. Lamellae brownish-tinged when mature; pileus with lilac to purplish tint; stipe violet-brown; basidiospores6–8 × 3–4.5 μm
*P. yuezhuoi*
9. Lamellae lilac-, vinaceous-, purplish- or violet-tinged1010. Pileus pink to lilac pink; lamellae pink; stipe whitish to pale pink; smell and taste strongly raphanoid; basidiospores 7–8.5 × 4–5 μm
*P. roseus*
10. Pileus and lamellae without strong pink tint1111. Stipe pinkish to reddish-brown; pileus purplish to dark purplish; lamellae pale lilac; basidiospores 5.5–7 × 3–4.5 μm
*P. densilamellatus*
11. Stipe lilac-, vinaceous-, purplish- or violet-tinged1212. Pleurocystidia present; pileus lilac- to violet-tinged; lamellae pale to pale violet; stipe pale, pinkish, violet to pinkish purple; basidiospores 6–9.5 × 4–5.2 μm
*P. purus*
12. Pleurocystidia absent1313. Basidiospore width up to less than 4.5 μm1413. Basidiospore width up to 5 μm or more1514. Pileus pale vinaceous to fawn; stipe lilac to reddish brown; basidiospores 6.8–8.3 × 3–4.3 μm
*P. subpurus*
14. Pileus cream, buff to lilac; stipe lilac to greyish violet; Basidiospores 5–8.3 × 3–4.3 μm
*P. variisporus*
15. Pileus pale vinaceous to lilac; lamellae pale vinaceous to livid vinaceous; stipe greyish violet to fuscous; basidiospores 6.7–8.2 × 3.8–5.3 μm
*P. violaceardesiacus*
15. Basidiomata without vinaceous tint1616. Pileus applanate, pale lilac to pale lilac brown; lamellae lilac white to lilac creamy; stipe lilac to pale purplish-pink; basidiospores ellipsoid, 6.5–8.4 × 4–5.5 μm, amyloid
*P. leptocollus*
16. Pileus pale rose, clay pink to pale violaceous; lamellae greyish violet at first, brownish with lilac tint at maturity; stipe dark violet with brownish tint at first, pale lilaceous brown to pale, dingy flesh-colored; basidiospores 6–9 × 3.5–5 μm, non-amyloid
*P. pearsonianus*
17. Basidiospore length up to less than 9 μm1817. Basidiospore length up to 9 μm or more2018. Basidiospore 5.4–7.1 × 2.6–4.0 μm, pileus brown; lamellae densely covered with dark brown dots, edge brown; stipe greyish-tinged
*P. brunneocystidiatus*
18. Basidiospore width up to 4.5 or more1919. Pileus pale pink to pale grey-brown; lamellae pale pinkish to whitish; stipe pale brown to grayish brown; basidiospores 5.5–8.5 × 3–4.5 μm
*P. applanatus*
19. Pileus dull red, greyish-brown, reddish brown and dark brown; lamellae white; stipe brownish orange, dull red, greyish brown to reddish brown; basidiospores 6.0–7.9 × 3.3–4.6 μm
*P. subulatus*
20. Basidiospores 6.7–9.1 × 3–4.2 μm; pileus cream, pinkish buff, buff-yellow to fawn; lamellae white to pinkish buff; stipe white, cream to pinkish buff
*P. subbrunneus*
20. Basidiospore width up to 4.5 μm or more2121. Pileus brown to dark brown, margin paler; lamellae yellowish gray to reddish gray; basidiospores 6.8–10.4 × 3.2–4.8 μm
*P. seminau*
21. Pileus brownish orange to yellowish brown, margin paler; lamellae orange-white to yellowish gray; basidiospores 7.2–9.6 × 3.2–4.8 μm
*P. sinar*



## Figures and Tables

**Table 1:** Primers used for PCR amplification.

Locus	Name	Primer Sequence	Reference
ITS	ITS5	5′-GGAAGTAAAAGTCGTAACAAGG-3′	White et al. (1990) [22]
	ITS4	5′-TCCTCCGCTTATTGATATGC-3′	
*rpb1*	RPB1Mp_f1	5′-AATTGGGGGAAACTGAAAGC-3′	Harder et al. (2013) [21]
	RPB1Mp_r1	5′-TGTCTCGCAGACCATCTTTG-3′	
*tef-1α*	tEFMp_f1	5′-TGGTGGTACTGGTGAGT-3′	Harder et al. (2013) [21]
	tEFMp_r1	5′-GGAAGACGGAGTGGCTTGT-3′	
	tEFMp_f2	5′-CTGGTGAGTTCGAAGCTGGT-3′	
	tEFMp_r2	5′-ACGTCCTGCAGGGGAAGAC-3′	

**Table 2:** DNA sequences of *Prunulus* used in the phylogenetic analysis in this study.

			GenBank Accession Numbers		
Species	Voucher	Locality	ITS	*rpb1*	*tef-1α*
*Mycena meliigena*	39d	Italy	JF908429	—	—
*M. metata*	HMJAU43625	USA	MH396636	—	—
***Prunulus applanatus***	**HMAS295877**	**China**	**PX674456**	**PX698948**	**PX675335**
***P. applanatus***	**HMAS297151, holotype**	**China**	**PX674455**	**PX698947**	—
***P. brunneocystidiatus***	**HMAS297156**	**China**	**PX674473**	**PX698963**	**PX675348**
***P. brunneocystidiatus***	**HMAS282091**	**China**	**PX674474**	**PX698964**	—
***P. brunneocystidiatus***	**HMAS300891**	**China**	**PX674472**	—	—
*P. brunneocystidiatus*	FFAAS3400, holotype	China	PV939239	—	—
*P. brunneocystidiatus*	FFAAS3401	China	PV939240	—	—
*P.* cf. *purus* VIII	CBH402	Denmark	FN394599	KF723663	KF723617
*P. densilamellatus*	TUFC101999, holotype	Japan	LC777686	LC777726	LC777734
*P. densilamellatus*	TUMH65482	Japan	LC777688	LC777728	LC777736
***P. fulvescens***	**HMAS303283**	**China**	**PX674470**	**PX698961**	**PX675346**
***P. fulvescens***	**HMAS303282, holotype**	**China**	**PX674471**	**PX698962**	**PX675347**
***P. fulvipes***	**HMAS286952, holotype**	**China**	**PX674464**	**PX698955**	**PX675340**
***P. fulvipes***	**HMAS292937**	**China**	**PX674465**	**PX698956**	**PX675341**
***P. fulvipes***	**HMAS297643**	**China**	**PX674466**	**PX698957**	**PX675342**
***P. fulvipes***	**HMAS292950**	**China**	**PX674467**	**PX698958**	**PX675343**
***P. fulvipes***	**HMAS293348**	**China**	**PX674468**	**PX698959**	**PX675344**
***P. fulvipes***	**HMAS294212**	**China**	**PX674469**	**PX698960**	**PX675345**
***P. fulvipes***	**HMAS291798**	**China**	**PX674462**	**PX698953**	—
***P. fulvipes***	**HMAS292372**	**China**	**PX674463**	**PX698954**	—
*P. lammiensis*	HMAS292295	China	OR236991	—	—
*P. lammiensis*	H6008503	Finland	MW540672	—	—
***P. leptocollus***	**HMAS300892**	**China**	**PX674457**	**PX698949**	**PX675336**
***P. leptocollus***	**HMAS300893**	**China**	**PX674458**	**PX698950**	**PX675337**
***P. leptocollus***	**HMAS294518, holotype**	**China**	**PX674459**	—	**PX675338**
***P. leptocollus***	**HMAS291678**	**China**	**PX674461**	**PX698952**	**PX675339**
***P. leptocollus***	**HMAS300895**	**China**	**PX674460**	**PX698951**	—
*P. pearsonianus*	CBH068	Germany	FN394614	KF723691	KF723645
*P. pearsonianus*	JV06890	Denmark	FN394612	KF723692	KF723646
*P. pearsonianus*	FCME25817, epitype	USA	JN182198	—	—
*P. pelianthinus*	CBH015	Denmark	FN394549	KF723695	KF723649
*P. pelianthinus*	CBH016	Denmark	FN394547	KF723696	KF723650
*P. polycystidiatus*	FFAAS0417, holotype	China	ON427731	ON468456	ON468469
*P. polycystidiatus*	FFAAS0421	China	ON427733	ON468458	ON468471
*P. purus*	IS10/11/2000	USA	FN394611	—	—
*P. roseus*	CBH409	Germany	FN394551	KF723683	KF723637
*P. roseus*	TL12393	Denmark	FN394555	KF723684	KF723638
*P. rufobrunneus*	FFAAS0415	China	ON427729	ON468454	ON468467
*P. rufobrunneus*	FFAAS0416, holotype	China	ON427730	ON468455	ON468468
*P. seminau*	ACL136	Malaysia	KF537250	—	—
*P. seminau*	ACL308	Malaysia	KF537252	—	—
*P. shengshanensis*	FFAAS0424, holotype	China	ON427739	ON468464	ON468477
*P. shengshanensis*	FFAAS0425	China	ON427740	ON468465	ON468478
*P. sinar*	ACL092	Malaysia	KF537247	—	—
*P. sinar*	ACL135	Malaysia	KF537249	—	—
*P. subbrunneus*	Liu265	China	PP037946	PP034076	PP034079
*P. subbrunneus*	Liu453, holotype	China	PP037951	PP034077	PP034082
*P. subpurus*	Liu10	China	PP037943	—	PP034083
*P. subpurus*	Liu489, holotype	China	PP037954	—	PP034084
*P. subulatus*	TUMH65484	Japan	LC777691	LC777731	LC777739
*P. subulatus*	FFAAS0423, holotype	China	ON427737	ON468462	ON468475
*P. variisporus*	Liu369, holotype	China	PP037949	—	PP034086
*P. variisporus*	Liu370	China	PP037950	—	PP034087
*P. violaceardesiacus*	Liu475	China	PP037952	—	PP034088
*P. violaceardesiacus*	Liu477, holotype	China	PP037953	—	—
*P. yuezhuoi*	FFAAS0345, holotype	China	MW581491	MW868169	MW882250
*P. yuezhuoi*	FFAAS0347	China	MW581493	MW868167	MW882252

Notes: New sequences obtained in this study are displayed in bold; “—” denotes missing sequences.

## Data Availability

All sequences generated in this study have been deposited in the NCBI database, and the species descriptions have been registered in Fungal Names, with the corresponding accession numbers provided in the manuscript.
